# Nebivolol elicits a neuroprotective effect in the cuprizone model of multiple sclerosis in mice: emphasis on M1/M2 polarization and inhibition of NLRP3 inflammasome activation

**DOI:** 10.1007/s10787-022-01045-4

**Published:** 2022-08-10

**Authors:** Antoinette G. Naeem, Reem N. El-Naga, Haidy E. Michel

**Affiliations:** Department of Pharmacology and Toxicology, Faculty of Pharmacy, Ain Shams University, Cairo, Egypt

**Keywords:** Multiple sclerosis, Cuprizone, Nebivolol, NLRP3, M1/M2 polarization

## Abstract

**Background and Aim:**

Multiple sclerosis (MS) is a demyelinating neurodegenerative inflammatory disease affecting mainly young adults. Microgliosis-derived neuroinflammation represents a key hallmark in MS pathology and progression. Nebivolol (Neb) demonstrated antioxidant, anti-inflammatory and neuroprotective properties in several brain pathologies. This study was conducted to investigate the potential neuroprotective effect of Neb in the cuprizone (Cup) model of MS.

**Methods:**

C57Bl/6 mice were fed 0.2% Cup mixed into rodent chow for 5 weeks. Neb (5 and 10 mg/kg/day) was administered by oral gavage during the last 2 weeks.

**Results:**

Neb prevented Cup-induced weight loss and motor deficits as evidenced by increased latency to fall in the rotarod test and enhanced locomotor activity as compared to Cup-intoxicated mice. Neb reversed Cup-induced demyelination as confirmed by Luxol fast blue staining and myelin basic protein western blotting. Administration of Neb modulated microglial activation status by suppressing M1 markers (Iba-1, CD86, iNOS, NO and TNF-α) and increasing M2 markers (Arg-1 and IL-10) as compared to Cup-fed mice. Furthermore, Neb hindered NLRP3/caspase-1/IL-18 inflammatory cascade and alleviated oxidative stress by reducing lipid peroxidation, as well as increasing catalase and superoxide dismutase activities.

**Conclusion:**

These findings suggest the potential neuroprotective effect of Neb in the Cup-induced model of MS in mice, at least partially by virtue of shifting microglia towards M2 phenotype, mitigation of NLRP3 inflammasome activation and alleviation of oxidative stress.

## Introduction

Multiple sclerosis (MS) is a demyelinating inflammatory disease of the central nervous system (CNS) representing the main cause of non-traumatic disability in young adults. The etiology of MS is not well understood. However, it may be related to viral infections, genetic predisposition, and many environmental factors (Dobson and Giovannoni 2019). Epidemiological studies have shown that MS has an estimated worldwide prevalence of 2.8 million people (Walton et al. 2020). The pathophysiology of MS is complex; including peripheral T-cells infiltration into the CNS, oligodendrocytes apoptosis, microgliosis, and neuroinflammation leading to demyelination and axonal injury (Lassmann 2014a).

Microgliosis plays an essential role in neuroinflammation and demyelination (Lassmann 2014b). Microglia, the CNS macrophages, are present in two main phenotypes: the classical pro-inflammatory M1 phenotype and the alternatively activated anti-inflammatory M2 phenotype (Xu et al. 2015). The M1 microglia exacerbate inflammation by producing cytotoxic mediators; such as tumor necrosis factor-alpha (TNF-α) and reactive oxygen species (ROS). By contrast, the M2 microglia release anti-inflammatory protective mediators such as interleukin 10 (IL-10) and hence promote tissue repair (Mayer et al. 2016). The nucleotide-binding oligomerization domain-like (NOD-like) receptor pyrin-containing 3 inflammasome (NLRP3) is a multimeric protein complex involved in amplifying inflammatory signals by the maturation of pro-inflammatory IL-18 (Shen et al. 2018). The serum and active lesions of MS patients show higher IL-18 levels (Cannella and Raine 2004; Keane et al. 2018). Inhibiting the NLRP3 inflammasome pathway was shown to inhibit microglial activation status in vitro by suppressing M1 polarization (Ślusarczyk et al. 2018). All the aforementioned data suggest that suppressing the NLRP3 inflammasome is a potential pharmacological target for alleviating microgliosis and neuroinflammation.

Demyelination is experimentally induced by the copper chelator, cuprizone (Cup) (Matsushima and Morell 2001). Feeding of 0.2% (w/w) Cup mixed into rodent chow for 5–6 weeks has been widely used to produce consistent demyelination in many brain areas; including the largest myelinated tract: the corpus callosum (CC) (Hiremath et al. 1998; Goldberg et al. 2015). Demyelination is evident after 3 weeks of Cup feeding (Vega-Riquer et al. 2019). The copper-dependent mitochondrial enzyme, cytochrome c oxidase, is inhibited by Cup leading to oligodendrocytes apoptosis and accumulation of myelin debris triggering recruitment of microglia that produce excess pro-inflammatory cytokines thus exacerbating demyelination as reviewed (Sen et al. 2019).

Nebivolol (Neb), a third-generation β-blocker, is an anti-hypertensive drug with distinct profile compared to other β-blockers due to its vasodilatory effect through the nitric oxide (NO) pathway (Kamp et al. 2010). It has been recognized as a strong antioxidant and anti-inflammatory drug (El-Sheikh et al. 2019). Furthermore, Neb can cross blood–brain barrier (BBB) (Prisant 2008) and provides neuroprotection against cerebral ischemia/reperfusion injury (Heeba and El-Hanafy 2012), reserpine-induced neuro-behavioral alterations (Nade et al. 2013) and cisplatin-induced depressive-like behavior (Abdelkader et al. 2017) in rats. Wang et al. showed that Neb reduces amyloid neuropathology in a mouse model of Alzheimer’s disease (Wang et al. 2013). It has been proven that Neb attenuates inflammation and microglial activation by decreasing pro-inflammatory cytokines secretion and NLRP3 inflammasome activation (Xie et al. 2016; Gao et al. 2019).

Accordingly, the present study was conducted to assess the potential neuroprotective effect of Neb in the Cup model of MS and to characterize the potential mechanism with respect to demyelination, oxidative stress, neuroinflammation and microgliosis in addition to its effect on the NLRP3 inflammasome pathway.

## Material and methods

### Animals

Thirty male C57Bl/6 mice, weighing 20–25 g, were purchased from Theodor Bilharz research institute (Giza, Egypt). Mice were housed in plastic cages at constant temperature (21 ± 2 °C) and under a 12 h light/dark cycle. Animals were acclimated for 2 weeks before starting the experiment and were provided access to rodent chow and water ad libitum. Body weight was measured weekly during the experiment. Animal handling strictly complied with institutional and international guidelines concerning the care and use of laboratory animals and complied with the National Institutes of Health guide for the care and use of laboratory animals (NIH Publications No. 8023, revised 1978). The experimental protocol was evaluated and approved by the research ethics committee of the Faculty of Pharmacy, Ain Shams University, Cairo, Egypt (Approval number: 274, December 2019).

### Drugs and chemicals

Cuprizone [Bis(cyclohexanone) oxaldihydrazone] was purchased from Fluka, Sigma-Aldrich Co. (USA). Nebivolol was obtained as a generous gift from Marcyrl Pharmaceutical industries (Cairo, Egypt). Carboxymethyl cellulose (CMC) was purchased from El-Nasr Pharmaceutical Company (Cairo, Egypt). Nebivolol was suspended in 0.5% CMC aqueous solution and vortexed thoroughly until a uniform suspension was obtained. All other chemicals were of the highest pure grade commercially available.

### Experimental design

**Fig. 1:**
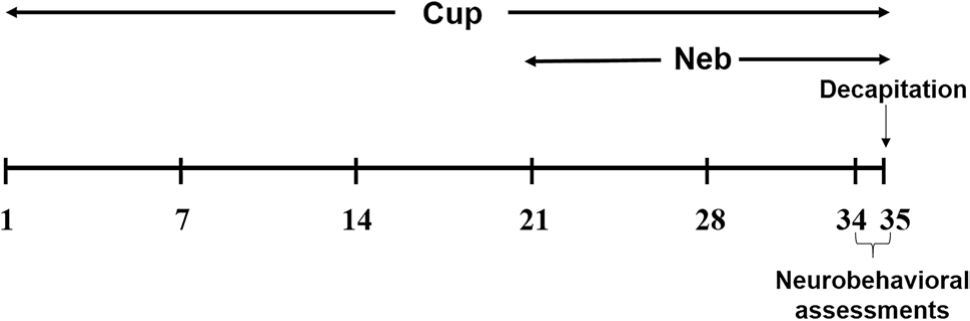
Study timeline showing Cup and Neb dosing regimens

Animals were randomly assigned into one of 5 groups (*n* = 6):

The first group served as a control group and received standard rodent chow for 5 weeks and the vehicle (0.5% CMC) during the last 2 weeks. The second group received a 0.2% Cup diet for 5 weeks plus oral gavage of 0.5% CMC during the last 2 weeks. The third and fourth groups received a 0.2% Cup diet for 5 weeks plus oral gavage of Neb (5 and 10 mg/kg/day, respectively) suspended in 0.5% CMC during the last 2 weeks (Baumhäkel et al. 2008). The fifth group received standard rodent chow for 5 weeks, in addition to an oral daily dose of 10 mg/kg Neb in 0.5% CMC during the last 2 weeks (Fig. 1).

On day 35, animals were subjected to neurobehavioral tests; rotarod test and locomotor activity assessment. Then, animals were euthanized by cervical dislocation; whole brains were excised, and the two hemispheres were separated. One hemisphere was stored at− 80 °C for biochemical analyses and the other hemisphere was fixed in 10% neutral buffered formalin for 72 h for histopathological examination.

### Neurobehavioral assessment

#### Rotarod test

Motor coordination and balance were evaluated using the rotarod apparatus. Mice were trained to walk on a rotating rod with increasing speed from 4 to 35 rpm over 3 min. On the next day, decreased ability to remain on the rotating rod (latency to fall) over 5 min was recorded (Chang et al. 2017).

#### Locomotor activity

Locomotor activity of mice was assessed using an activity monitor (Opto-Varimex-Mini Model B, Columbus Instruments, Columbus, OH, USA). Mice (*n* = 6) were allowed 1 min habituation in the recording chamber, then, the frequency of interruption of 15 infrared rays (*λ* = 875 nm, scan rate = 160 Hz, diameter = 0.32 cm, and spacing = 2.65 cm) was recorded over 5 min in the dark. The locomotor activity of animals was expressed as counts/5 min (Zhang et al. 2012; Chang et al. 2017).

#### Histopathological examination

Histopathological examination was conducted by an independent researcher who was blind to the animal treatments. Briefly, brain samples were flushed and fixed in 10% neutral buffered formalin for 72 h. Samples were trimmed, processed in serial grades of ethanol, cleared in xylene then infiltrated and embedded into paraplast wax tissue embedding media. Four micrometer-thick tissue sections were cut by rotatory microtome for the demonstration of CC total area and mounted on glass slides from different samples. For Luxol fast blue (LFB) staining, the sections were deparaffinized, then rinsed in 100% ethanol and 95% ethanol, and then incubated in an LFB solution (0.01% in 95% ethanol) overnight at 60 °C. Whole brain sections were processed in 0.05% lithium carbonate solution, differentiated in 70% ethanol and counterstained with periodic acid–Schiff (PAS) stain, and then examined microscopically to detect cortical demyelination/remyelination. Six random non-overlapping fields were captured from the CC in six sections from each group (6 mice/group) for the determination of the positive area percent of myelinated nerve fibers in CC regions stained by LFB. All micrographs were obtained using a full HD microscopic camera operated by the Leica application module (Leica Microsystems GmbH, Wetzlar, Germany). Image analysis was performed using ImageJ software (version 1.50i) (Drury,and Wallington 1983).

#### Assessment of oxidative and nitrosative stress markers

Brain lipid peroxidation levels were measured colorimetrically as thiobarbituric acid reactive substances (TBARS) using kits purchased from Biodiagnostic (Giza, Egypt) according to the manufacturer’s instructions. Results were expressed as nmol malondialdehyde (MDA)/ml. Antioxidant catalase (CAT) and superoxide dismutase (SOD) enzymes’ activities in the brain were assessed using colorimetric kits purchased from Biodiagnostic (Giza, Egypt) according to the manufacturer’s instructions. Results were expressed as unit/mg protein. Brain NO level was quantified as nitrite and measured colorimetrically using a kit obtained from Biodiagnostic (Giza, Egypt) according to the manufacturer’s instructions. Results were expressed as nmol nitrite/mg protein.

#### Assessment of tumor necrosis factor alpha, interleukin 10 and interleukin 18

Tumor necrosis factor alpha (TNF-α), IL-10 and IL-18 were measured in brain homogenate using a sandwich enzyme immunoassay technique utilizing ELISA kits according to the manufacturer’s instructions. TNF-α and IL-18 ELISA kits were purchased from Elabscience (Houston, USA), while IL-10 ELISA kit was purchased from Cloud-Clone Corporation (Houston, USA). Concentration was expressed as pg/mg protein.

#### Western blot

Brain whole tissue lysates were prepared using RIPA buffer standard protocol (Bio Basic Inc, Markham, Ontario, Canada). Then, samples were centrifuged, and protein quantification was performed using Bradford Protein Assay Kit (Bio Basic Inc, Markham, Ontario, Canada). Protein (20 μg) was loaded per well of a 10% SDS-PAGE gel using electrophoresis buffer, separated, and then transferred onto a PVDF membrane (Bio-Rad Laboratories, Hercules, CA, USA). Membrane blocking was done using TBST and 5% BSA for 1 h followed by overnight incubation with one of the following primary antibodies (1:1000): myelin basic protein (MBP) (CAT # ab155995; Abcam, Cambridge, MA, USA), arginase-1 (Arg-1) (CAT # 93,668; Cell Signaling Technology, Danvers, MA, USA), ionized calcium-binding adapter molecule 1 (Iba-1) (CAT # 17,198; Cell Signaling Technology, Danvers, MA, USA), cluster of differentiation 86 (CD86) (CAT # ab112490; Abcam, Cambridge, MA, USA), inducible nitric oxide synthase (iNOS) (CAT # ab136918; Abcam, Cambridge, MA, USA), NLRP3 (CAT # AG-20B-0014, AdipoGen, San Diego, CA, USA) or cleaved caspase-1 (CAT # 89,332; Cell Signaling Technology, Danvers, MA, USA). Afterwards, the membranes were rinsed and incubated with secondary goat anti-rabbit IgG HRP-linked antibody solution (1: 5000) against the blotted target protein for 1 h at room temperature. Development was done using Clarity ™ Western ECL chemiluminescent substrate (Bio-Rad Laboratories, Hercules, CA, USA). The signal was captured using a Chemi Doc MP imager (Bio-Rad Laboratories, Hercules, CA, USA). Load correction was done using anti-β-actin polyclonal antibody (1:1000; CAT# 4970; Cell Signaling Technology, Danvers, MA, USA). Quantification of band densities was done using imageJ software (version 1.50i) (Gallo-Oller et al. 2018).

#### Statistical analysis

Statistical analysis was implemented using GraphPad Prism software (version 9, ISI^®^ software, USA). Normality test was performed using D’Agostino-Pearson normality test. Bartlett’s test was performed to test for homogeneity of variances. Since the data proved to be normally distributed and have homogenous variances, parametric tests were employed. Body weight results were analyzed using two-way ANOVA followed by Bonferroni post hoc test. Multiple comparisons for all other parameters were performed using one-way ANOVA followed by the Tukey post hoc test. Data were presented as mean ± standard deviation (SD). Inter-group statistical significance was considered at *p* value less than 0.05.

## Results

### Neb improved cup-induced decrease in locomotor activity

**Fig. 2:**
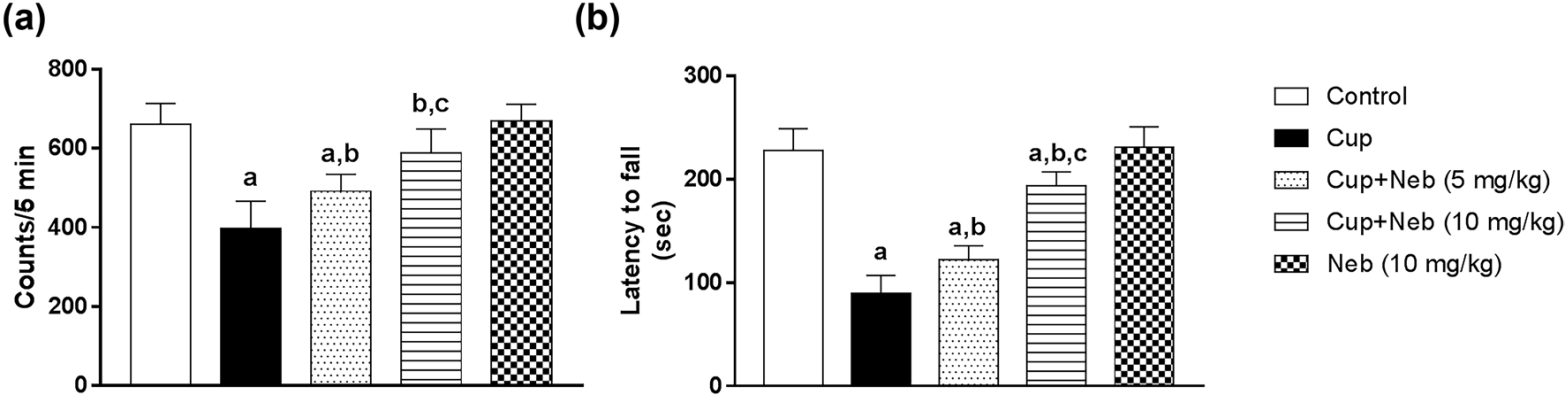
The effect of Neb on Cup-induced motor abnormalities. **a** Locomotor activity test. **b** Rotarod test. Data are presented as means ± SD (*n* = 6). **a**, **b**, **c**: Statistically significant from the control, Cup and Cup + Neb (5 mg/kg)-treated groups, respectively, at *P* < 0.05. Statistical analysis was performed using one-way ANOVA followed by Tukey’s test for multiple comparisons between groups

Administration of Cup for 35 days induced a significant decrease in the locomotor activity by 39.95%, as compared to the control group (*p* < 0.0001) (Fig. 2a). Co-treatment with Neb (5 and 10 mg/kg) significantly increased the locomotor activity in comparison to Cup-intoxicated animals by 1.23 (*p* < 0.05) and 1.48-fold (*p* < 0.0001), respectively (Fig. 2a).

### Neb enhanced cup-induced changes in motor coordination and balance

Rotarod test showed that Cup-intoxicated animals spent less time on rotarod as evidenced by the statistically significant decrease in the latency to fall by 60.58%, compared to the control group (*p* < 0.0001) (Fig. 2b). However, Neb (5 and 10 mg/kg)-treated animals showed statistically marked increase in the latency to fall by 1.36 (*p* < 0.05) and 2.15-fold (*p* < 0.0001), respectively, as compared to Cup-intoxicated animals (Fig. 2b).

### Neb reduced cup-induced body weight loss

**Fig. 3:**
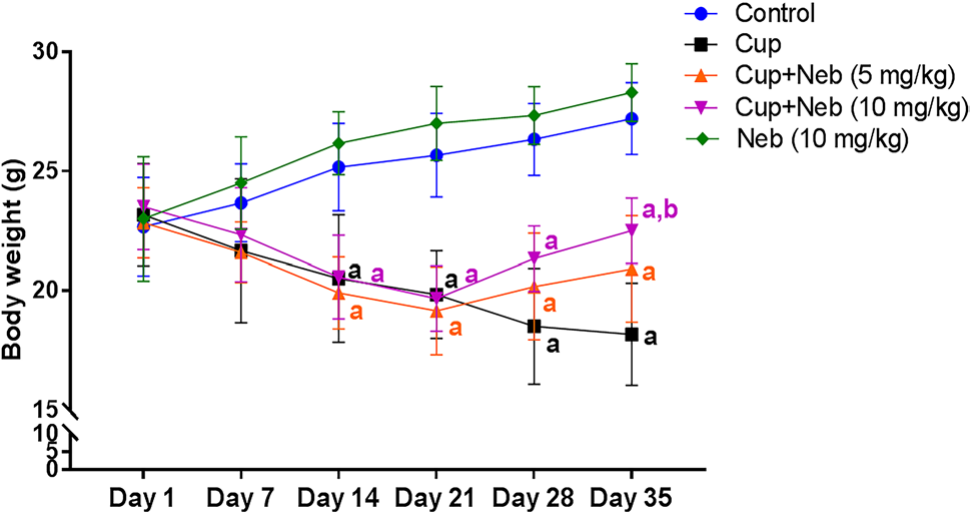
The effect of Neb on Cup-induced body weight changes. **a**, **b**:Statistically significant from the control and Cup-treated groups, respectively, at *P* < 0.05. Statistical analysis was performed using two-way ANOVA followed by Bonferroni test for multiple comparisons between groups

As shown in Fig. 3, no significant difference in body weight was detected in all experimental groups for the first 13 days of Cup administration. On day 14, two-way ANOVA revealed that Cup-treated mice showed a significant body weight loss as compared to the control group (*p* < 0.001). Consumption of Cup from day 14 to day 35 led to further body weight loss as evidenced by a significant decline in body weight, compared to the control group (*p* < 0.0001). Treatment with Neb started from day 21 till day 35. The first week of Neb treatment resulted in increase in body weight, as compared to the Cup-treated group; however, this difference did not reach statistical significance for both doses. On the other hand, the last week of co-treatment with Neb (10 mg/kg) reduced the significant weight loss of Cup-exposed mice (*p* < 0.001). However, the increase in body weight of mice co-treated with Neb (5 mg/kg) did not reach statistical significance.

### Neb attenuated cup-induced demyelination

**Fig. 4:**
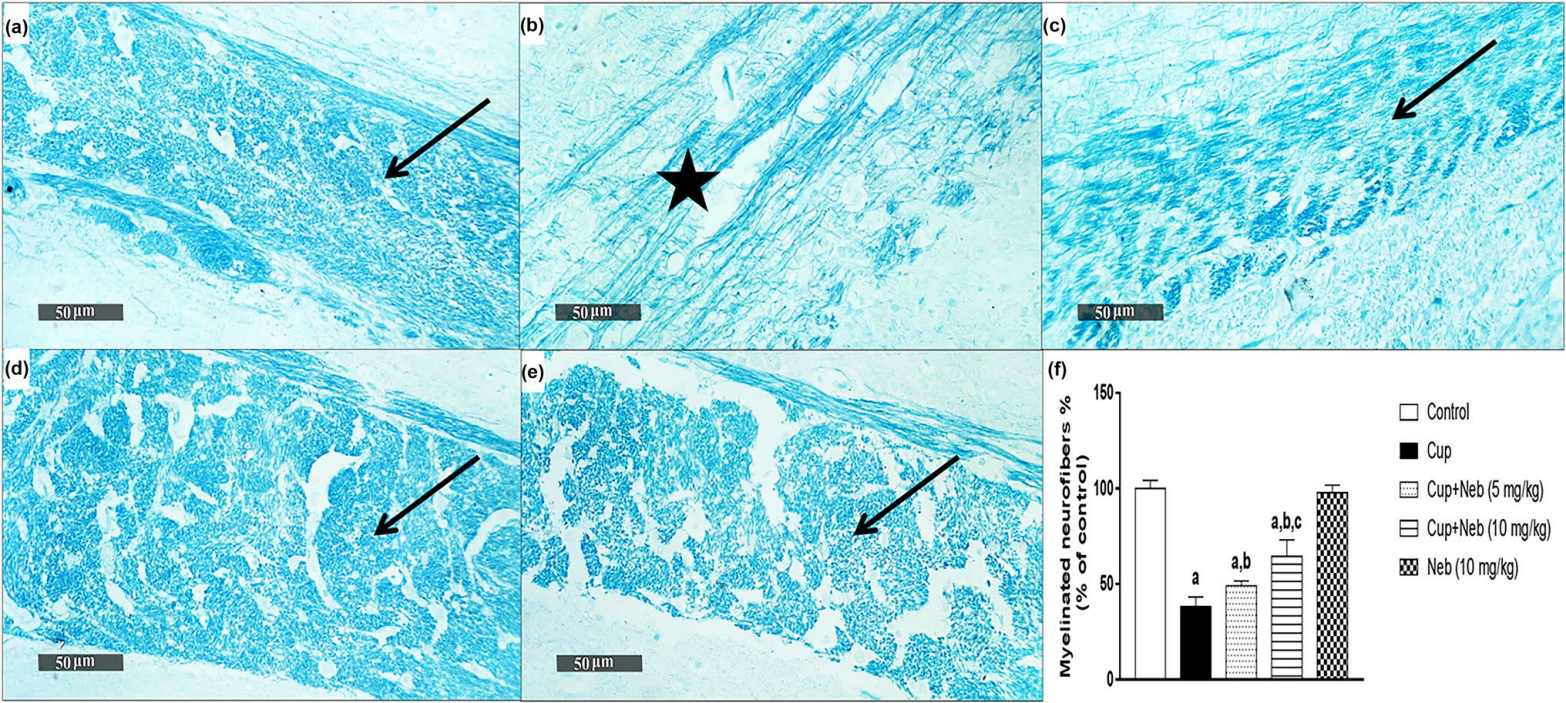
**a** Representative photomicrograph of LFB stained CC sections (Control group) showing normal positive staining intensity (arrow). **b** Cup group showing a significant decrease in staining intensity (star). **c**, **d** Cup + Neb (5 and 10 mg/kg), respectively, both showing elevated staining intensity as compared to Cup-intoxicated mice (arrow). **e** Neb (10 mg/kg) showing positive staining intensity (arrow). Magnification 400× . **f** Percentage of myelinated neurofibers resulting from LFB expressed as a percentage of control. Data are presented as means ± SD (*n* = 6). **a**, **b**, **c**: Statistically significant from the control, Cup and Cup + Neb (5 mg/kg)-treated groups, respectively, at *P* < 0.05. Statistical analysis was performed using one-way ANOVA followed by Tukey’s test for multiple comparisons between groups

**Fig. 5:**
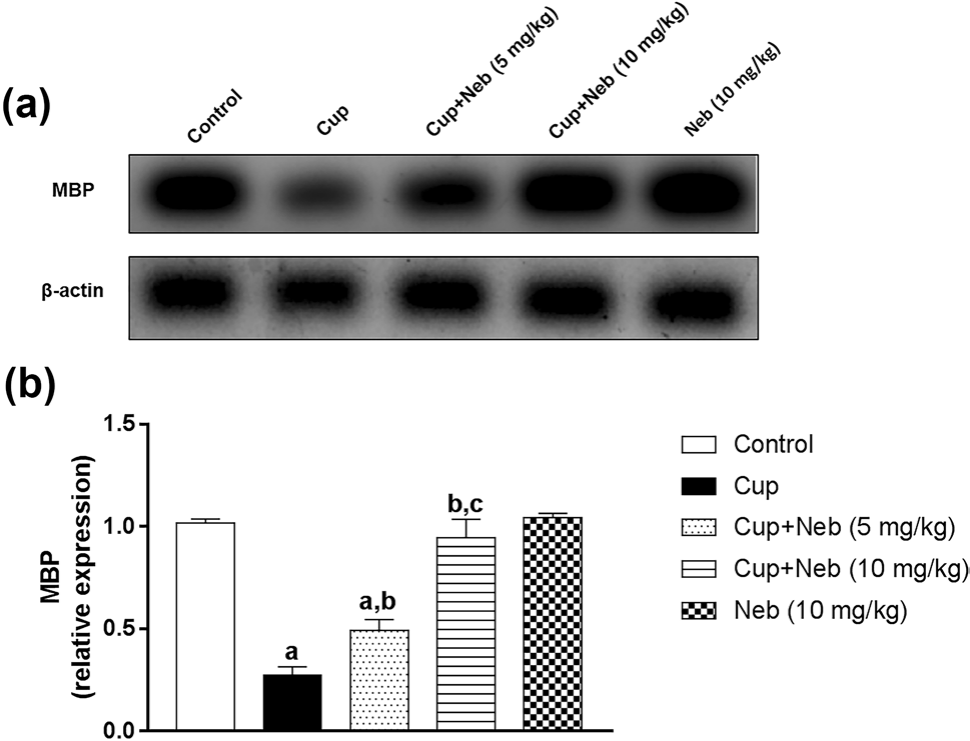
**a** Western blot analysis of brain MBP. **b** Densitometric quantitation of MBP protein expression. Data are presented as means ± S.D. (*n* = 3). **a**, **b**, **c**: Statistically significant from the control, Cup and Cup + Neb (5 mg/kg)-treated groups, respectively, at *P* < 0.05. Statistical analysis was performed using one-way ANOVA followed by Tukey's test for multiple comparisons between groups

Myelin status was assessed by LFB staining of the CC and western blotting of MBP. Significant demyelination was detectable in Cup-intoxicated mice as shown by a decrease in the intensity of LFB staining and the percentage of myelinated neurofibers by 61.85%, compared to the control group (*p* < 0.0001) (Fig. 4). Co-treatment with Neb (5 and 10 mg/kg) resulted in increase in the intensity of LFB staining and the percentage of myelinated neurofibers by 1.28 (*p* < 0.05) and 1.69-fold (*p* < 0.0001), respectively. In addition, Cup-intoxication decreased the protein expression levels of MBP by 73.13% (*p* < 0.0001), in comparison to the control group (Fig. 5). However, co-treatment with Neb (5 and 10 mg/kg) upregulated MBP expression by 1.79 (*p* < 0.01) and 3.45-fold (*p* < 0.0001), respectively, indicating that Neb protected against Cup-induced demyelination.

### Neb attenuated microglial activation and suppressed M1 polarization in cup-intoxicated mice

**Fig. 6:**
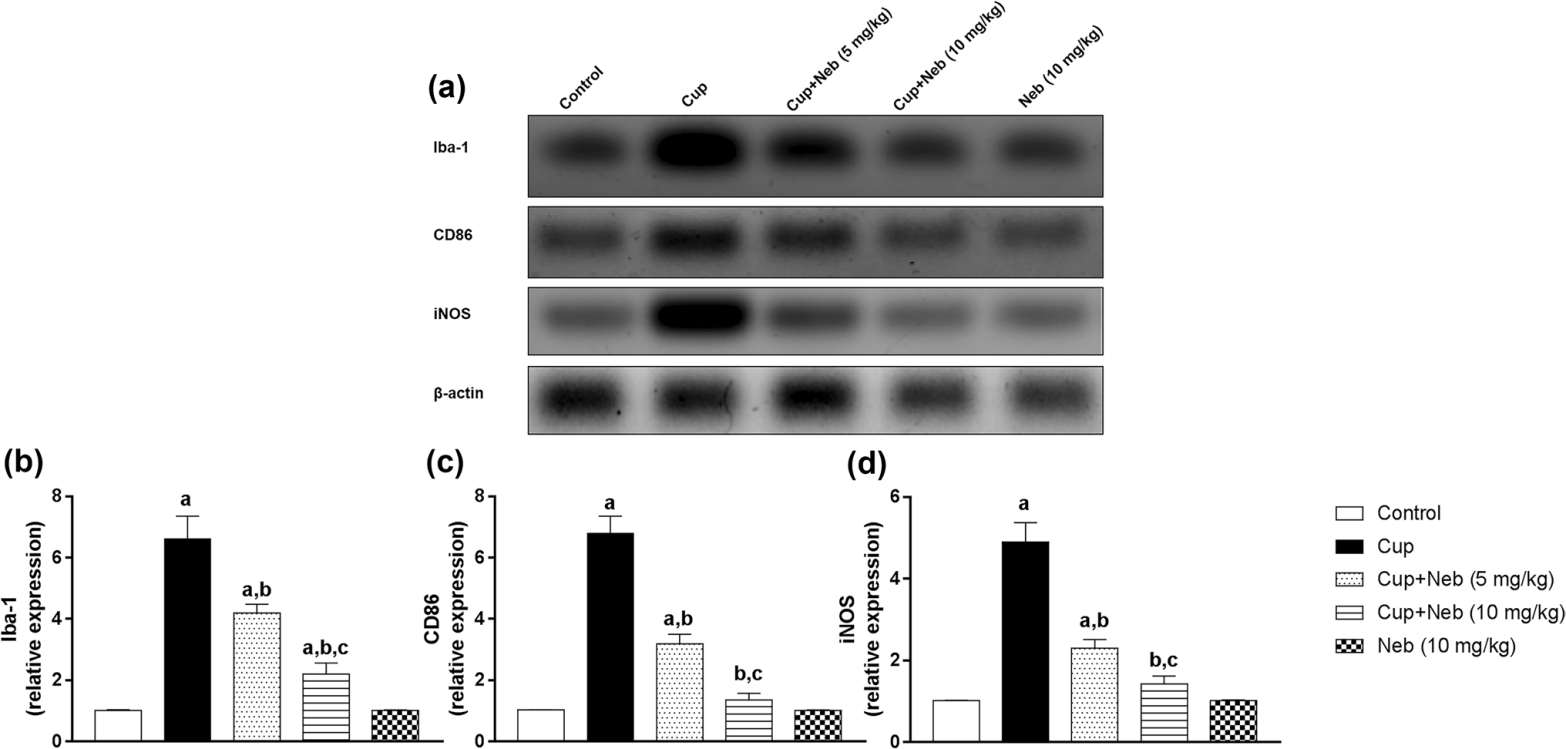
**a** Western blot analysis of brain Iba-1, CD86 and iNOS. **b**, **c** and **d** Densitometric quantitation of Iba-1, CD86 and iNOS protein expression, respectively. Data are presented as means ± SD. (*n* = 3). **a**, **b**, **c**: Statistically significant from the control, Cup and Cup + Neb (5 mg/kg)-treated groups, respectively, at *P* < 0.05. Statistical analysis was performed using one-way ANOVA followed by Tukey’s test for multiple comparisons between groups

**Fig. 7:**
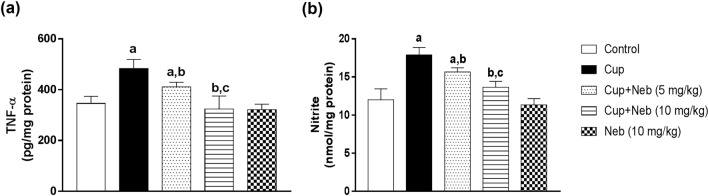
The effect of Neb on TNF-α (**a)** and nitrite (**b)** levels in the brain tissues of Cup-treated mice. Data are presented as means ± SD (*n* = 6). **a**, **b**, **c**: Statistically significant from the control, Cup and Cup + Neb (5 mg/kg)-treated groups, respectively, at *P* < 0.05. Statistical analysis was performed using one-way ANOVA followed by Tukey’s test for multiple comparisons between groups

The protein expression of the microglial activation marker; Iba-1, was significantly increased by 6.42-fold (*p* < 0.0001) in Cup-intoxicated mice compared to the control group while Neb (5 and 10 mg/kg) administration significantly downregulated Iba-1 level by 36.51% (*p* < 0.001) and 66.67% (*p* < 0.0001), respectively, in comparison to Cup-treated mice. M1 polarization was evaluated by assessing M1 markers: CD86, iNOS, NO and TNF-α levels. The protein expression of CD86 and iNOS was significantly higher in Cup-intoxicated mice by 6.6 and 4.8-fold (*p* < 0.0001), respectively, as compared to the control group. Treatment with Neb (5 mg/kg) resulted in a significant reduction of the protein expression of CD86 and iNOS by 52.94% and 53.03%, respectively, (*p* < 0.0001), as compared to Cup-treated mice. Similarly, Neb (10 mg/kg)-treated mice exhibited a significant decline in CD86 and iNOS protein expression levels by 79.89% and 71.02%, respectively, (*p* < 0.0001), in comparison to Cup-treated mice (Fig. 6). The levels of TNF-α and NO were significantly elevated in Cup-intoxicated animals by 1.39 and 1.48-fold, respectively, compared to the control group (*p* < 0.0001). Treatment with Neb (5 mg/kg) significantly reduced the levels of TNF-α by 14.96% and NO by 12.41% (*p* < 0.01), as compared to Cup-treated mice. Moreover, Neb (10 mg/kg) significantly reduced the levels of TNF-α and NO by 32.88% and 23.75%, respectively, (*p* < 0.0001), as compared to Cup-treated mice (Fig. 7).

### Neb promoted M2 polarization in cup-intoxicated mice

**Fig. 8:**
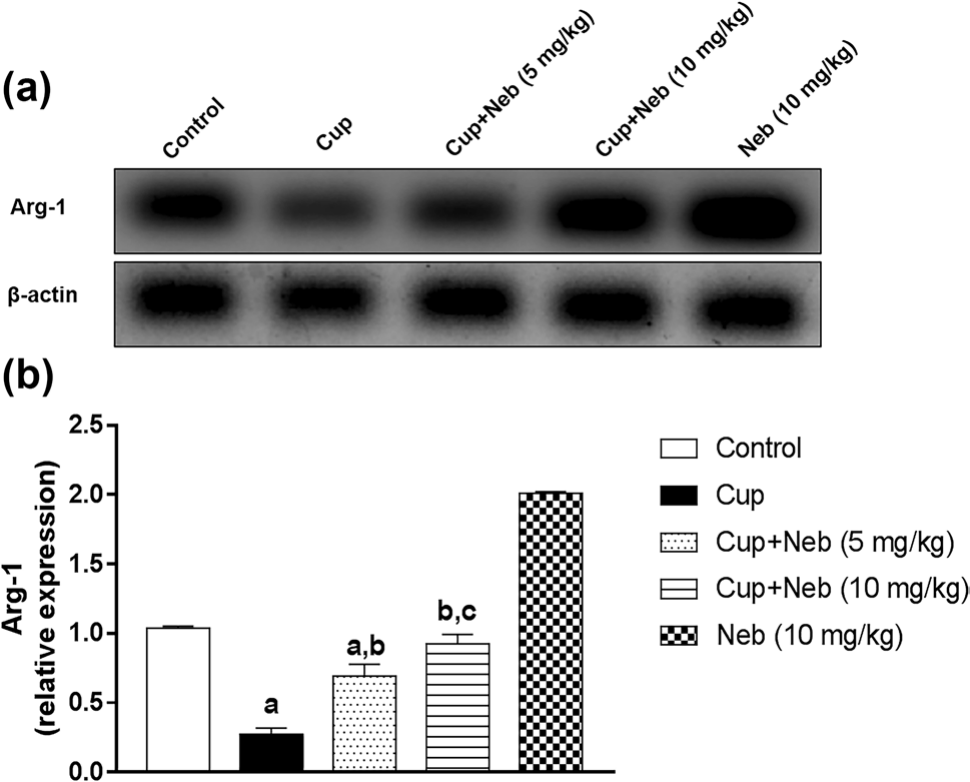
**a** Western blot analysis of brain Arg-1. **b** Densitometric quantitation of Arg-1 protein expression. Data are presented as means ± S.D. (*n* = 3). **a**, **b**, **c**: Statistically significant from the control, Cup and Cup + Neb (5 mg/kg)-treated groups, respectively, at *P* < 0.05. Statistical analysis was performed using one-way ANOVA followed by Tukey’s test for multiple comparisons between groups

**Fig. 9:**
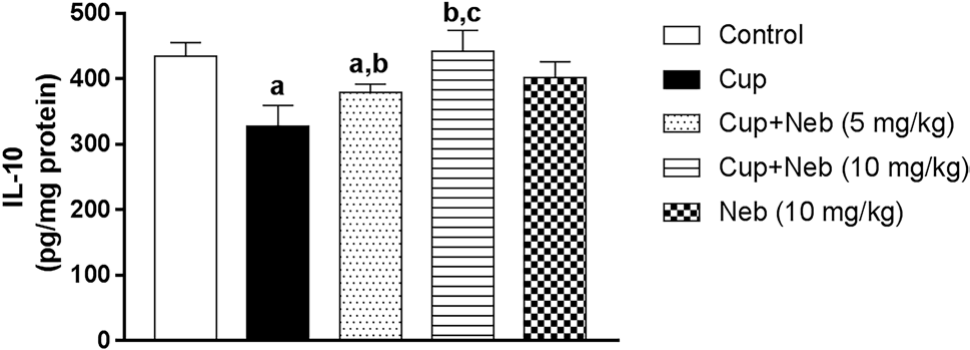
The effect of Neb on IL-10 levels in the brain tissues of Cup-treated mice. Data are presented as means ± SD (*n* = 6). **a**, **b**, **c**: Statistically significant from the control, Cup and Cup + Neb (5 mg/kg)-treated groups, respectively, at *P* < 0.05. Statistical analysis was performed using one-way ANOVA followed by Tukey's test for multiple comparisons between groups

M2 polarization was analyzed using M2 markers; Arg-1 and IL-10. As shown in Fig. 8, statistically significant decrease in the protein expression levels of Arg-1 was observed in Cup-treated mice by 73.54% (*p* < 0.0001), as compared to the control. In contrast, Neb (5 and 10 mg/kg) upregulated Arg-1 protein expression by 2.54 and 3.38-fold, respectively, (*p* < 0.0001), in comparison to Cup-intoxicated mice. Interestingly, Cup also reduced IL-10 levels by 24.61% compared to the control group (*p* < 0.0001). However, co-treatment with Neb (5 and 10 mg/kg) increased IL-10 levels by 1.16 (*p* < 0.05), and 1.35- fold (*p* < 0.0001), respectively, in comparison to Cup-treated mice (Fig. 9).

### Neb counteracted NLRP3 inflammasome activation in cup-treated mice

**Fig. 10:**
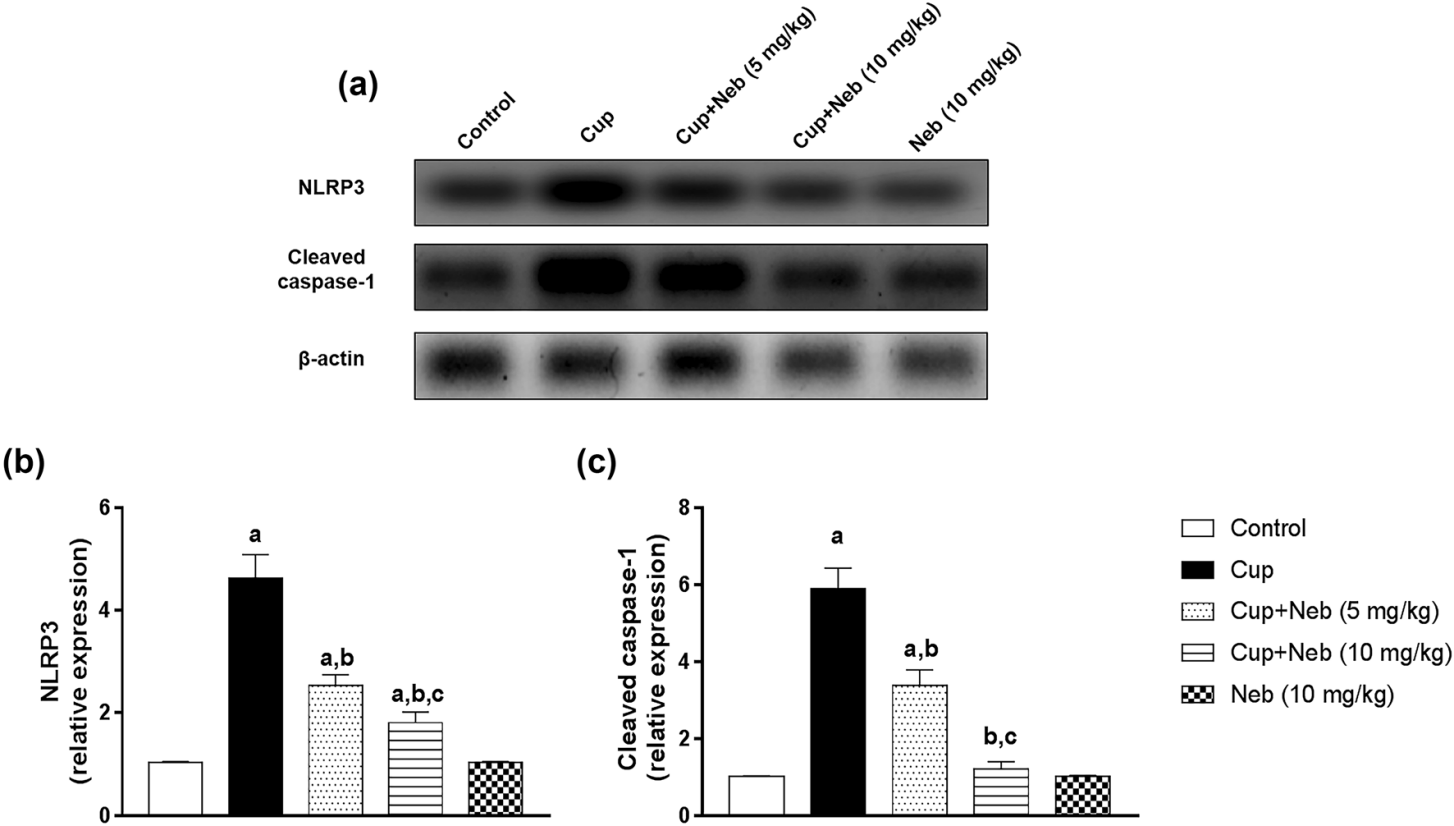
**a** Western blot analysis of brain NLRP3 and cleaved caspase-1. **b** Densitometric quantitation of NLRP3 protein expression. **c** Densitometric quantitation of cleaved caspase-1 protein expression. Data are presented as means ± SD. (*n* = 3). **a**, **b**, **c**: Statistically significant from the control, Cup and Cup + Neb (5 mg/kg)-treated groups, respectively, at *P* < 0.05. Statistical analysis was performed using one-way ANOVA followed by Tukey’s test for multiple comparisons between groups

**Fig. 11:**
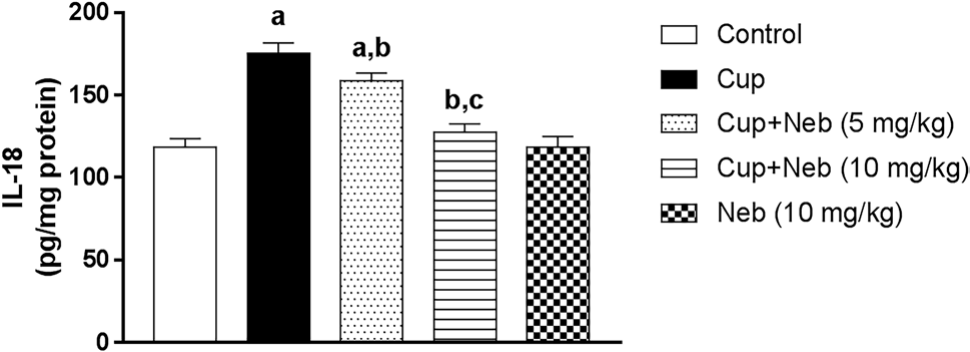
The effect of Neb on IL-18 levels in the brain tissues of Cup-treated mice. Data are presented as means ± SD (*n* = 6). **a**, **b**, **c**: Statistically significant from the control, Cup and Cup + Neb (5 mg/kg)-treated groups, respectively, at *P* < 0.05. Statistical analysis was performed using one-way ANOVA followed by Tukey’s test for multiple comparisons between groups

The effect of Neb on NLRP3 inflammasome activation was evaluated by assessment of the protein expression levels of NLRP3 and cleaved caspase-1 (Fig. 10) in addition to the level of its end-product; IL-18 (Fig. 11). Administration of Cup upregulated the protein expression levels of NLRP3 and cleaved caspase-1 by 4.47 and 5.78-fold, respectively, as well as IL-18 level by 1.48-fold, compared to the control group (*p* < 0.0001). Co-treatment with Neb (5 mg/kg) suppressed NLRP3 activation as evidenced by a decrease in NLRP3 and cleaved caspase-1 protein expression levels by 45.33% and 42.93%, respectively, (*p* < 0.0001), and IL-18 levels by 9.41% (*p* < 0.001), in comparison to Cup-treated animals. Moreover, treatment of Cup-administered mice with Neb (10 mg/kg) downregulated NLRP3 and cleaved caspase-1 protein levels by 61% and 79.49%, respectively, as well as IL-18 level by 27.21%, (*p* < 0.0001).

### Neb alleviated cup-induced oxidative stress

**Fig. 12:**
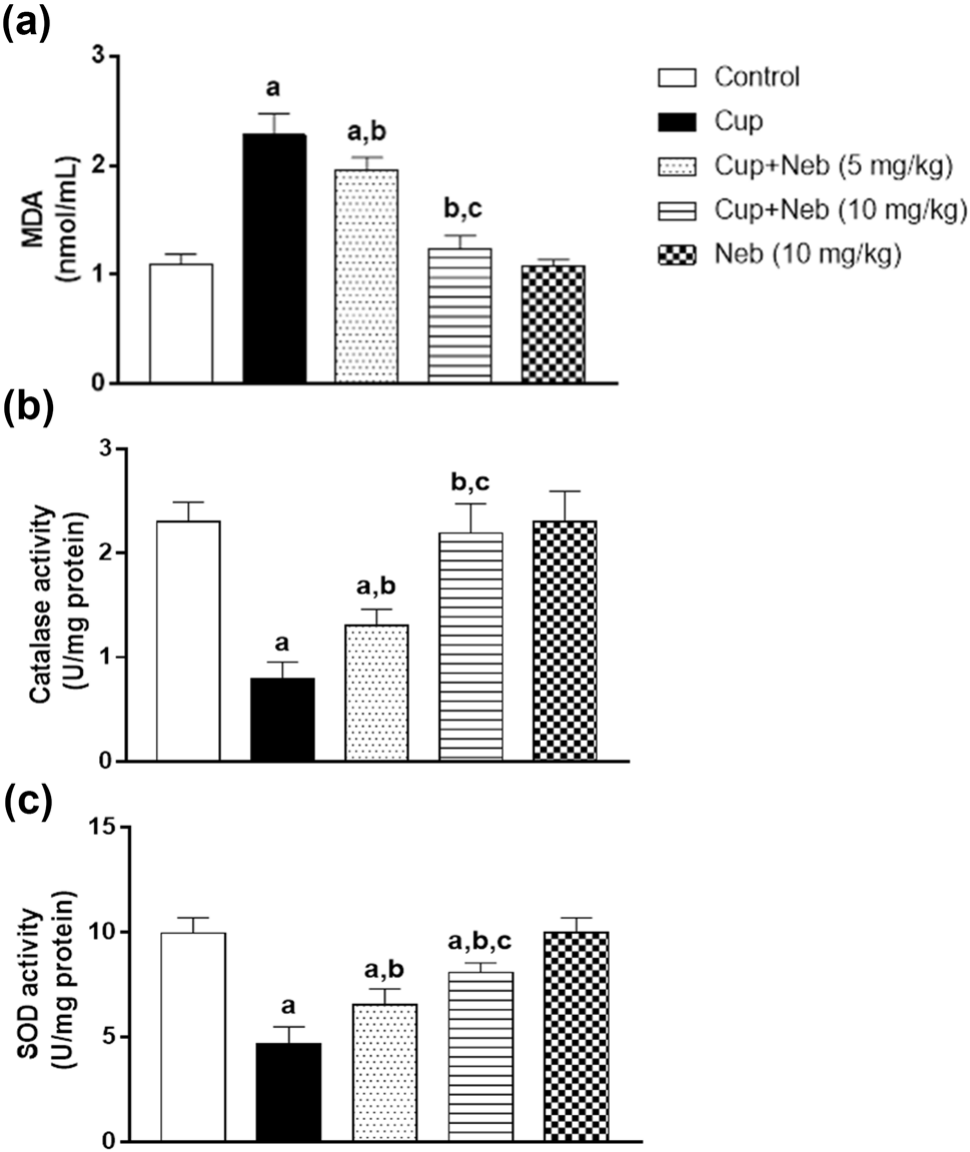
The effect of Neb on oxidative stress markers: MDA levels **a**, catalase **b** and SOD **c** activities in the brain tissues of Cup-intoxicated mice. Data are presented as means ± SD (*n* = 6). **a**, **b**, **c**: Statistically significant from the control, Cup and Cup + Neb (5 mg/kg)-treated groups, respectively, at *P* < 0.05. Statistical analysis was performed using one-way ANOVA followed by Tukey’s test for multiple comparisons between groups

Oxidative stress was determined by measuring MDA levels in addition to catalase and SOD activities. Administration of Cup resulted in a significant elevation in MDA level by 2.08-fold, when compared to the control group (*p* < 0.0001) (Fig. 12a). Administration of Neb (5 mg and 10 mg/kg) resulted in a statistically significant decline in MDA levels by 14.12% (*p* < 0.01) and 45.65% (*p* < 0.0001), respectively, as compared to Cup-treated mice. Catalase activity was significantly reduced by 65.62% (*p* < 0.0001) in Cup-intoxicated animals in comparison to the control group. Noteworthy, Neb (5 and 10 mg/kg) administration significantly ameliorated Cup-induced decrease in catalase activity by 1.65-fold (*p* < 0.01) and 2.76-fold (*p* < 0.0001), respectively, in comparison to Cup-treated mice (Fig. 12b). Likewise, Cup-treated mice exhibited a statistically significant decrease in SOD activity by 52.77%, as compared to the control animals (*p* < 0.0001). On the other hand, Neb (5 and 10 mg/kg)-treated mice showed a statistically significant elevation in SOD activity by 1.39-fold (*p* < 0.01) and 1.71-fold (*p* < 0.0001), respectively, in comparison to Cup-administered mice (Fig. 12c).

## Discussion

The present study aimed to investigate the potential neuroprotective effect of Neb in the Cup model of MS and to elucidate the possible underlying mechanisms. Our findings showed that Neb administration significantly reversed Cup-induced demyelination, motor abnormalities, and weight loss via downregulating NLRP3 inflammasome signaling, promoting M2 polarization, and alleviating oxidative stress, suggesting the neuroprotective effect of Neb against Cup-induced model of MS in mice.

Sustained neuroinflammation involving microglia represents a key pathological hallmark in MS (Fani Maleki and Rivest 2019). Counteracting MS neuroinflammatory demyelination by targeting microglial M1/M2 phenotypic shift and NLRP3 inflammasome pathway has received growing attention as evidenced by many studies (Aryanpour et al. 2017; Barati et al. 2019; Zhang et al. 2021). The Cup model highly reflects the neuroinflammatory demyelinating aspects of MS pathology. In addition, Cup induces demyelination without altering the BBB integrity ensuring no involvement of the peripheral inflammatory cells (Bakker and Ludwin 1987; Gudi et al. 2014). Hence, inflammatory mediators and ROS are mainly produced by microglia making the Cup model ideal for evaluating the effect of Neb on microgliosis and its deleterious role in demyelination.

Microglia, the main CNS immune cells, are implicated in CNS development and homeostasis as well as in clearing debris (Ginhoux et al. 2013). Disturbance of CNS homeostasis leads to microglial activation for protection against tissue damage. However, chronic activation in neurodegenerative diseases evokes a state of excessive microglial activation causing a cycle of inflammation and neurotoxicity rather than resolving the damage (London et al. 2013). On the other hand, the neuroprotective role of microglia in MS has been well-documented; microglia phagocytize myelin debris, recruit oligodendrocytes progenitor cells and release regenerative factors for remyelination (Napoli and Neumann 2010). This dual role of microglia is explained by the fact that they exist as two activation patterns; classical pro-inflammatory M1 activation and alternative anti-inflammatory M2 activation (Mayer et al. 2016). New therapeutic strategies should suppress harmful effects of aberrant microglial activation while retaining microglial physiological neuroprotective functions thus selective M2 polarization rather than complete microglial inhibition should be our target (Du et al. 2017).

M1 microglial activation is associated with excessive production of pro-inflammatory cytokines, such as TNF-α as well as enzymes; such as iNOS for NO production. This is in addition to the upregulation of cell surface molecules; such as CD86 (David and Kroner 2011; Orihuela et al. 2016). On the other hand, M2 microglial activation results in the release of anti-inflammatory cytokines; such as IL-10 and enzymes; such as Arg-1 for tissue repair (David and Kroner 2011). Our study showed that Cup intoxication significantly increased M1 markers levels (TNF-α, iNOS, NO and CD86) and suppressed M2 markers levels (IL-10 and Arg-1). This finding comes in line with many reports proving that M1 polarization contributes to Cup-induced inflammatory cascade (Aryanpour et al. 2017; Barati et al. 2019). Neb treatment significantly decreased the levels of M1 markers and augmented the levels of M2 markers. It was previously shown that Neb can suppress M1 polarization by reducing the density of macrophages expressing the M1 marker CD68 (Pyka-Fosciak et al. 2013). To the authors’ knowledge, this is the first study to demonstrate that Neb promotes M2 polarization which strengthens the promising modulatory effect of Neb on microglia-derived neuroinflammation.

iNOS and Arg-1 are markers of M1 and M2 polarization, respectively (David and Kroner 2011) with a common substrate (arginine) but different products making iNOS a potent inducer of inflammation, that is counteracted by Arg-1 (Kuo 1998; Aryanpour et al. 2017). Excessive expression of iNOS by activated microglia and associated high levels of NO is a hallmark of MS lesions (Smith and Lassmann 2002). Various mechanisms explain the deleterious role of NO in demyelination. The current study showed that Cup-induced increase in iNOS protein expression and NO levels were coincident with low Arg-1 protein expression. This effect was attenuated by the Neb administration. The decrease of NO levels by Neb may be attributed to the fact that iNOS is not significantly expressed in the CNS unless inflammation occurs (Hanisch and Kettenmann 2007; Ghasemi and Fatemi 2014). Hence, lower NO levels were detected in Neb-treated mice, by virtue of regulation of microglial phenotypic switching and subsequent reduction in M1-associated iNOS expression. This comes in line with another study which showed that Neb reduced NO levels in cisplatin-induced nephrotoxicity (Morsy and Heeba 2016).

Additionally, Iba-1 is a microglial surface protein which is widely used to assess all microglia regardless of their phenotype (Ito et al. 1998; Walker and Lue 2015). In our demyelinating model, Cup significantly increased Iba-1 protein expression, while co-treatment with Neb significantly suppressed it which further support our data indicating alleviation of neuroinflammation and microgliosis.

NLRP3 inflammasome is a multimeric protein complex involved in sensing danger signals. It is composed of sensor protein NLRP3, apoptosis-associated speck-like (ASC) adaptor protein and executor enzyme pro-caspase-1. The NLRP3 inflammasome complex formation triggers processing pro-IL-1β and pro-IL-18 into their active forms (Shen et al. 2018). Over-activation of NLRP3 inflammasome activation is implicated in MS pathogenesis as evidenced by elevated levels of caspase-1 and IL-18 in MS patients serum and cerebrospinal fluid (CSF) (Losy and Niezgoda 2001; Keane et al. 2018). Many studies showed that NLRP3 regulates microglial phenotypic switching suggesting that inhibition of NLRP3 inflammasome represents a potential strategy for alleviating neuroinflammation (Heneka et al. 2013; Liu et al. 2018; Cui et al. 2020).

The potential pathological implication of the NLRP3/caspase-1/IL-18 pathway in Cup-induced demyelination is suggested by increased NLRP3 levels in Cup-fed animals, compared to the control. In addition, Jha et.al demonstrated that the knockout of NLRP3, caspase-1 or IL-18 gene delayed Cup-induced demyelination and neuroinflammation (Jha et al. 2010). In accordance, our study showed that Neb significantly decreased NLRP3 and cleaved caspase-1 protein expression as well as IL-18 levels in Cup-fed mice compared to untreated Cup-fed animals, highlighting the anti-inflammatory effect of Neb. This further confirms the modulatory effect of Neb on microglial activation status previously suggested by the current study as the suppression of NLRP3 inflammasome and its downstream inflammatory mediators contributed to the alleviation of microgliosis. This finding is in line with previous studies that demonstrated the inhibitory effect of Neb on NLRP3 inflammasome in a rat model of obesity-induced myocardial toxicity (Xie et al. 2016) and vascular remodeling (Gao et al. 2019).

In the present study, Cup-intoxicated mice exhibited motor abnormalities as evidenced by decreased locomotor activity and latency to fall in the rotarod test in agreement with previous studies (Ghaiad et al. 2017; Abd El Aziz et al. 2021). This is explained by the fact that Cup-induced CC demyelination impairs bilateral motor coordination (Liebetanz and Merkler 2006). Furthermore, Cup led to body weight loss in line with many previous studies (Hashimoto et al. 2017; Elbaz et al. 2018). Co-treatment with Neb resulted in significant improvement in motor performance in addition to reversing Cup-induced body weight loss. These results may be related to Neb antioxidant and anti-inflammatory effects demonstrated in this study.

The MBP is an essential myelin protein involved in the assembly and compaction of myelin sheath layers (Fulton et al. 2010). In the present study, Cup decreased MBP gene expression in accordance with the results of other studies (Chen et al. 2014; Abdel-Maged et al. 2020) as Cup chelates copper which is essential in myelin compaction resulting in myelin destabilization and degeneration (Frid et al. 2015). However, we showed, for the first time, that Neb treatment significantly increased MBP expression, as compared to the Cup group. This finding is in line with the histopathological examination which showed increased LFB staining intensity and the percentage area of myelinated fibers. Hence, Neb treatment reversed Cup-induced changes in myelin status due to its antioxidant, anti-inflammatory and modulatory effect on microglia activation shown in this study.

The copper-chelating agent, Cup, chelates the copper of the mitochondrial enzyme cytochrome c oxidase hence disturbing the electron transport chain leading to mitochondrial dysfunction and excessive generation of ROS resulting in the reduction of the antioxidant enzymes such as catalase and SOD (Ghaiad et al. 2017). In addition, it was found that Cup increases MDA levels in the corpus callosum leading to cell death (Largani et al. 2019). Myelin is characterized by a high lipid-to-protein ratio essential for myelin-packed structure (Williams et al. 1993) which makes myelin prone to oxidative damage. In our study, Cup intoxication-induced oxidative stress as shown by elevated MDA levels and decreased catalase and SOD enzymatic activities which is consistent with previous studies (Shiri et al. 2020). Administration of Neb alleviated Cup-induced oxidative stress by decreasing MDA levels and increasing catalase and SOD enzymatic activities. These findings are consistent with previous studies proving antioxidant properties of Neb (Nade et al. 2013; Imbaby et al. 2014; Refaie et al. 2018; El-Sheikh et al. 2019).

In conclusion, the current study investigated the potential neuroprotective effect of Neb in the Cup model of MS. We demonstrated that oral administration of Neb significantly counteracted Cup-induced demyelination, motor impairment and weight loss. In addition, Neb alleviated oxidative stress, attenuated neuroinflammation and microgliosis by modulating the microglial activation state and suppressing the NLRP3 inflammasome pathway. It is worth noting that, according to the FDA body surface area conversion (Food and Drug Administration 2005; Nair and Jacob 2016), the tested Neb doses are close to the clinically used doses with reported safety and tolerability (Fongemie and Felix-Getzik 2015). Taken together, Neb might prove beneficial in the management of MS.

## Data Availability

The datasets generated during and/or analysed during the current study are available from the corresponding author on reasonable request.
